# Pregnancy disorders in Africa and the obstetric dilemma

**DOI:** 10.1093/trstmh/trx005

**Published:** 2017-03-07

**Authors:** Annettee Nakimuli, Ashley Moffett

**Affiliations:** a Department of Obstetrics and Gynaecology, School of Medicine, Makerere University College of Health Sciences, Mulago Hill Road, P.O.Box 7072, Kampala, Uganda; b Department of Pathology, University of Cambridge, Cambridge, UK; c Centre for Trophoblast Research, Cambridge, UK

**Keywords:** Africa, Great Obstetrical Syndromes, KIR, Obstetric dilemma, Pre-eclampsia, Uganda

## Abstract

The high neonatal and maternal morbidity and mortality associated with the extremes of birth weight is referred to as the obstetric dilemma. Pre-eclampsia and other conditions that lead to low birth weight are considered as the Great Obstetrical Syndromes (GOS). At the other extreme is high birth weight resulting in obstructed labour. Fetal weight largely depends on placental function and defective placentation is a common feature of the GOS. There is evidence that the local uterine immune system (KIR and HLA-C) regulates placentation, with racial differences noted. These differences may be responsible for the striking obstetric dilemma in Africans.

The high rates of maternal and neonatal mortality in Sub-Saharan Africa remain high despite the efforts geared towards their reduction.^1^ The reasons for this are complex with a range of cultural, social, educational and political issues contributing. In all populations across the world the pregnancies most at risk from death and morbidity for both mothers and babies are those where the birth weight is at the extremes of the birth weight spectrum, a concept known as the obstetric dilemma.^2^ When the baby is very small and has failed to grow in utero there is a risk of death from stillbirth, prematurity, infection and failure to thrive. Because the feto-placental unit becomes stressed due to the poor nutrient and oxygen supply, this may trigger the syndrome of pre-eclampsia in the mother. All these conditions are considered as the Great Obstetrical Syndromes (GOS).^3^ At the other extreme, when the babies are large there is a risk of obstructed labour, haemorrhage, birth asphyxia and trauma to both mother and baby.

A range of genetic, nutritional and environmental factors regulate birth weight in humans with a genetic contribution from both parents. Our work has focussed on immune system genes because the placenta is the interface between two genetically different individuals, the mother and her fetus who is carrying paternal genes. The immune system can recognise and respond to polymorphic genes from the father analogous to the situation of allorecognition in transplantion. However, unlike transplantation, natural killer (NK) cells in the uterus mediate allorecognition of the fetus by the mother using receptors known as killer immunoglobulin-like receptors (KIR) that can distinguish between variants of HLA-C molecules displayed by the placental cells that invade the uterus. NK cells use an array of activating and inhibitory receptors to distinguish virally infected and cancerous cells from normal healthy cells.^4^ Because uterine NK cells use exactly the same KIR receptors to distinguish between fetal and maternal fetal cells it appears there are two strong contrasting evolutionary pressures: disease resistance and successful reproduction, both showing evidence of balancing selection.^2,5^ Genetic and functional studies give rise to the hypothesis that uterine NK cells regulate trophoblast transformation of the uterine spiral arteries necessary for increasing the blood supply to the feto-placental unit.^6,7^

Different KIR recognise two epitopes of all HLA-C allotypes known as C1 and C2 groups. The KIR family of genes are also highly polymorphic and closely located in KIR haplotypes.^8^ Consistent findings in UK and African cohorts are that certain combinations of maternal KIR genotypes with fetal HLA-C groups are associated with pre-eclampsia and other GOS.^9,10^ We have found that women who have a KIR AA genotype are at risk of pre-eclampsia/GOS when there is a paternal HLA-C allele bearing a C2 epitope. The frequency of such C2 alleles is much higher in Africa than in other populations, possibly one reason contributing to the higher risk of the GOS in Sub-Saharan Africa.^11^ How these genetic findings translate into changes in the functional response of the uterine NK cells is under investigation but the KIR A haplotype is characterised by the presence of a strongly inhibitory KIR for C2, KIR2DL1. In contrast the KIR B haplotype has a variable number of activating KIR, including KIR2DS1 that also binds to the C2 epitope. This fits with the general idea that NK cells function on the basis of the overall input from inhibitory and activating receptors and suggest that a very strong inhibitory signal from a paternal C2 results in defective placentation and failure of the trophoblast cells to transform the arteries to deliver enough oxygen and nutrients right until the end of gestation.^6^ In keeping with this idea, we have also found that European women who have activating KIR2DS1 and a KIR B haplotype are protected from pre-eclampsia and are more likely to have a baby with a high birth weight.^12^ Our findings in a Ugandan cohort were not able to replicate the protective effect of KIR2DS1 which is generally at very low frequency in Africans. Instead we found a unique centromeric KIR B region containing an enigmatic activating KIR, KIR2DS5, which is restricted to individuals with African ancestry.^10^ Furthermore, only certain alleles of KIR2DS5 showed protection, highlighting the importance of the recent genotyping methods developed in the field,^13^ which should result in more studies interrogating the allelic variation in KIR in disease association. These genetic findings in pre-eclampsia in Ugandans highlight the importance of studying a disease such as pre-eclampsia in the population that is particularly affected by it. Overall, the recent findings from genetic association studies suggest too much inhibition, likely driven by KIR2DL1 the potent HLA-C2 receptor, is detrimental to pregnancy. On the other hand, strong activation which could be driven by the HLA-C2 receptor, KIR2DS1 or potentially by KIR2DS5, have opposite effects. Activation can help balance inhibition and protect against GOS and small birth weight whereas too much activation could lead to high birth weight complications such as obstructed labour. What remains to be investigated is how allelic variability of KIR2DL1 and KIR2DS1 play a role and if the alleles of KIR2DS5, responsible for the effects observed in the Ugandan population, are functional HLA-C2 activating receptors.

The lack of exact replication of our findings come from the difficulties encountered by new groups trying to set up KIR and HLA typing and the lack of easily accessible statistical tools to analyse these unconventional datasets.^14^ Furthermore, KIR and HLA frequencies highly vary between populations explaining why different effects can be observed for the same underlying disease association.

KIR A and KIR B haplotypes and HLA-C alleles bearing C1 and C2 epitopes are present in all human populations–albeit at different frequencies–and are under balancing selection^5^ Keeping the birth weight between the two dangerous extremes is a classic example of balancing selection.^15^ This does suggest that maternal KIR and fetal (paternal) HLA-C variants may be under selection from the problems epitomised by the obstetric dilemma. However, these are immune system genes and are likely to also play a role in response to infections.^15^ This intersection between two systems, response to infectious disease and reproductive success is interesting. Why there is a higher frequency of HLA-C alleles bearing the C2 epitope in Sub-Saharan Africa is unknown but C2 might provide a beneficial role in certain infections. A combination of infectious disease selection and reproductive selection seems to have driven the evolution of KIR B-like haplotypes from a KIR A-like founder haplotype.^15^ Continued selection to survive and to reproduce maintains a balance between KIR A and KIR B as well as C1 and C2 HLA-C groups in all populations. Also intriguing is the low frequency of KIR2DS1 in Sub-Saharan Africa and the presence of KIR2DS5 that could reflect the out of Africa migration of early modern humans and the subsequent admixture with Neanderthals.

In conclusion, the long held belief that immune cells must be suppressed for successful pregnancy–both locally in the uterus and systemically–that originated with Medawar and the birth of transplant biology needs a reappraisal to focus instead on the role of NK cell allorecognition using two highly polymorphic gene families, maternal KIR and fetal HLA.
